# Retraction: Overexpression of microRNA-132 enhances the radiosensitivity of cervical cancer cells by down-regulating Bmi-1

**DOI:** 10.18632/oncotarget.28413

**Published:** 2023-05-04

**Authors:** Gui-Feng Liu, Shu-Hua Zhang, Xue-Feng Li, Li-Yan Cao, Zhan-Zhao Fu, Shao-Nan Yu

**Affiliations:** ^1^Department of Radiology, China-Japan Union Hospital of Jilin University, Changchun 130033, P.R. China; ^2^Operating Room, China-Japan Union Hospital of Jilin University, Changchun 130033, P.R. China; ^3^Department of Anesthesiology, China-Japan Union Hospital of Jilin University, Changchun 130033, P.R. China

**This article has been retracted:** In Figure 8, panel B, three of the tumor images are duplicates of images published in three other journals [1–3]. In addition, multiple internal image duplications occurred in Figure 4. The authors are unable to recover the original data and cannot verify the accuracy of their conclusions. As a result, all authors have agreed to the retraction of this paper from Oncotarget.

Original article: Oncotarget. 2017; 8:80757–80769. 80757-80769. https://doi.org/10.18632/oncotarget.20358
